# Renormalized vibrations and normal energy transport in 1d FPU-like discrete nonlinear Schrödinger equations

**DOI:** 10.1038/s41598-018-23719-2

**Published:** 2018-03-28

**Authors:** Simeng Li, Nianbei Li

**Affiliations:** 1 Center for Phononics and Thermal Energy Science, School of Physics Science and Engineering, Tongji University, 200092 Shanghai, P. R. China; 2 Institute of Systems Science and Department of Physics, College of Information Science and Engineering, Huaqiao University, Xiamen, 361021 China

## Abstract

For one-dimensional (1d) nonlinear atomic lattices, the models with on-site nonlinearities such as the Frenkel-Kontorova (FK) and *ϕ*^4^ lattices have normal energy transport while the models with inter-site nonlinearities such as the Fermi-Pasta-Ulam-*β* (FPU-*β*) lattice exhibit anomalous energy transport. The 1d Discrete Nonlinear Schrödinger (DNLS) equations with on-site nonlinearities has been previously studied and normal energy transport has also been found. Here, we investigate the energy transport of 1d FPU-like DNLS equations with inter-site nonlinearities. Extended from the FPU-*β* lattice, the renormalized vibration theory is developed for the FPU-like DNLS models and the predicted renormalized vibrations are verified by direct numerical simulations same as the FPU-*β* lattice. However, the energy diffusion processes are explored and normal energy transport is observed for the 1d FPU-like DNLS models, which is different from their atomic lattice counterpart of FPU-*β* lattice. The reason might be that, unlike nonlinear atomic lattices where models with on-site nonlinearities have one less conserved quantities than the models with inter-site nonlinearities, the DNLS models with on-site or inter-site nonlinearities have the same number of conserved quantities as the result of gauge transformation.

## Introduction

The discrete translational symmetry conserves total momentum in solid crystals without on-site potentials. In 1d atomic lattices, the low frequency long-wavelength phonons are responsible for the anomalous energy transport discovered in momentum-conserved nonlinear lattices such as the FPU-*β* lattice with inter-site nonlinearities^1^. It has been argued that the nature of momentum conservation is the key issue for anomalous energy transport in 1d lattices^2–5^, until normal energy transport is found in the 1d coupled rotator lattice where total momentum is also conserved^6,7^. In contrast, the 1d nonlinear atomic lattices with on-site nonlinearities such as the FK and *ϕ*^4^ lattices do not conserve total momentum and have normal energy transport^8–12^. The coupled rotator lattice has been viewed as an exception as all the other 1d nonlinear atomic lattices with momentum conservation exhibit anomalous energy transport^13–23^. Most recently, the divergent thermal conductivities as the signature of anomalous energy transport has been experimentally verified for the 1d carbon nanotubes with a size up to 1 mm^24^.

The 1d DNLS equation is another kind of widely studied nonlinear lattice model which deals with the classical dynamics of a complex-valued field^25^. The DNLS model is a reasonable approximation for the wave evolution in nonlinear optical wave guides^26–29^, dilute Bose-Einstein condensates (BEC)^30,31^, and electronic transport in biomolecules^32^, to name a few. The nonintegrable 1d DNLS model can conserve both the total energy and the number of particles. The energy diffusion at zero temperature background is ballistic out of self-trapping regime due to soliton transport in ordered^33^ DNLS models. In disordered DNLS models, things are more complicated and whether the energy spreading appears or not is a matter of probability^34–38^. Recently, the energy transport at finite temperature for DNLS models has attracted much attention^39–42^. These pioneer works have arrived the results that the energy transport in DNLS models is normal for not too small nonlinearity or temperature. However, these mostly studied DNLS models are all with on-site nonlinearities whose atomic lattice counterparts are 1d nonlinear lattices with on-site nonlinearities such as the Frenkel-Kontorova (FK), *ϕ*^4^ and nonlinear Klein-Gordon (KG) lattices which exhibit normal energy transport^8–12^. It is thus not surprising to find normal energy transport in 1d DNLS models with on-site nonlinearities. How does the energy transport behave in 1d FPU-like DNLS models with inter-site nonlinearities remains to be a more interesting problem to be investigated.

In this article, we will study the dynamics of 1d FPU-like DNLS models with inter-site nonlinearities. The renormalized vibration theory valid for phonons will be developed for the 1d DNLS models and the existence of general renormalized vibrations will be verified by numerical simulations. The energy transport will be numerically studied through the energy diffusion processes in equilibrium states. It is found that the 1d FPU-like DNLS models exhibit *normal* energy transport, totally different from their atomic lattice counterparts such as the FPU-*β* lattice.

## Results

We consider the Hamiltonian of 1d FPU-like DNLS models with inter-site nonlinearities as the following^43^:

1 $$H=\sum _{j}\,[{g}_{2}{|{\psi }_{j+1}-{\psi }_{j}|}^{2}+{g}_{4}{|{\psi }_{j+1}-{\psi }_{j}|}^{4}]$$

where *ψ*_*j*_ denotes the complex-valued field at site *j*. The parameters *g*_2_ and *g*_4_ represent the coefficients of quadratic and quartic interactions, respectively. The DNLS model is FPU-*β*-like with *g*_2_ = *g*_4_ = 1 and *H*_4_-like with *g*_2_ = 0 and *g*_4_ = 1. The *H*_4_ atomic lattice is the high temperature limit of FPU-*β* lattice with pure nonlinear potential term. The equations of motion are generated by $id{\psi }_{j}/dt=\partial H/\partial {\psi }_{j}^{\ast }$ where ${\psi }_{j}^{\ast }$ is the complex conjugate of *ψ*_*j*_. The conserved quantities are the total energy of Eq. (1) and the norm (particle number) of *S* = ∑_*j*_|*ψ*_*j*_|^2^.

The linear DNLS model with *g*_4_ = 0 in Eq. (1) is an integrable system. The normal vibrational modes of linear DNLS model can be obtained by considering the travelling wave solution as *ψ*_*j*_ ∝ *e*^*i*(*kj*−*ωt*)^. One can derive the exact dispersion relation of the normal vibrational modes as

2 $${\omega }_{k}=4{g}_{2}{\sin }^{2}(\frac{k}{2})$$

where 0 ≤ *k* < 2*π* is the wave number. The normal vibrational modes are gapless since *ω*_*k*_ → 0 as *k* → 0. In the low frequency limit, the dispersion relation behaves as *ω*_*k*_ ∝ *k*^2^ which resembles the out-of-plane ZA phonon modes in 2D materials such as the Graphene^44^. However, the origin of this square behavior is due to the fact that the time derivative of the Schrödinger equation is first order.

The including of the nonlinear term (*g*_4_ > 0) in Eq. (1) introduces the interactions between normal vibrational modes. In particular, the nonlinear interactions in DNLS models will induce renormalized vibrations as that of atomic lattices. For atomic lattice systems, it is well established that nonlinear interactions will cause renormalized phonons^45–52^. For example, the FPU-*β* lattice with Hamiltonian $H={\sum }_{j}[{p}_{j}^{2}\mathrm{/2}+{({q}_{j+1}-{q}_{j})}^{2}\mathrm{/2}+{({q}_{j+1}-{q}_{j})}^{4}\mathrm{/4]}$ has renormalized phonon spectrum as ${\omega }_{k}^{R}=\sqrt{\alpha }{\omega }_{k}$, where ${\omega }_{k}=2\,\sin \,(k/2)$ is the exact phonon modes in Harmonic lattice of $H={\sum }_{j}[{p}_{j}^{2}/2+{({q}_{j+1}-{q}_{j})}^{2}/2]$. The renormalization coefficient *α* can be obtained as *α* = (〈(*q*_*j*+1_ − *q*_*j*_)^2^〉 + 〈(*q*_*j*+1_ − *q*_*j*_)^4^〉)/〈(*q*_*j*+1_ − *q*_*j*_)^2^〉, where 〈⋅〉 denotes ensemble average or time average in equivalence for ergodic systems and *α* is independent of lattice index *j*^45^.

Before we formulate the renormalized vibration theory for DNLS models, it is convenient to make the following canonical transformation ${\psi }_{j}=({q}_{j}+i{p}_{j})/\sqrt{2}$. The Hamiltonian of Eq. (1) becomes

3 $$\begin{array}{rcl}H({q}_{j},{p}_{j}) & = & \sum _{j}\,[{g}_{2}[\frac{1}{2}{({q}_{j+1}-{q}_{j})}^{2}+\frac{1}{2}{({p}_{j+1}-{p}_{j})}^{2}]\\  &  & \,+{g}_{4}{[\frac{1}{2}{({q}_{j+1}-{q}_{j})}^{2}+\frac{1}{2}{({p}_{j+1}-{p}_{j})}^{2}]}^{2}]\end{array}$$

where the real valued *q*_*j*_ and *p*_*j*_ are another set of generalized canonical coordinates and momenta, respectively. The quadratic term is linear part and the quartic term is nonlinear part. The dynamics of Eq. (3) is then determined by the usual Hamiltonian equations *dq*_*j*_/*dt* = ∂*H*/∂*p*_*j*_, *dp*_*j*_/*dt* = −∂*H*/∂*q*_*j*_.

Considering a reference linear DNLS model after renormalization as

4 $${H}^{R}=\sum _{j}\alpha [\frac{1}{2}{({q}_{j+1}-{q}_{j})}^{2}+\frac{1}{2}{({p}_{j+1}-{p}_{j})}^{2}]$$

where *α* is the renormalization coefficient to be determined. The generalized equipartition theorem states that 〈*q*_*j*_∂*H*/∂*q*〉 = *k*_*B*_*T* where *k*_*B*_ is the Boltzmann constant and *T* is the temperature. By requiring that 〈*q*_*j*_∂*H*/∂*q*_*j*_〉 = 〈*q*_*j*_∂*H*^*R*^/∂*q*_*j*_〉 for Hamiltonian of Eqs (3) and (4), one can derive the renormalized vibrations as

5 $$\begin{array}{rcl}{\omega }_{k}^{R} & = & 4\alpha \,{\sin }^{2}(\frac{k}{2})\\ \alpha  & = & {g}_{2}+2{g}_{4}\frac{\langle {[\frac{1}{2}{({q}_{j+1}-{q}_{j})}^{2}+\frac{1}{2}{({p}_{j+1}-{p}_{j})}^{2}]}^{2}\rangle }{\langle \frac{1}{2}{({q}_{j+1}-{q}_{j})}^{2}+\frac{1}{2}{({p}_{j+1}-{p}_{j})}^{2}\rangle }\end{array}$$

where the nonlinear information are incorporated into the renormalization coefficient *α*. The renormalized vibrations represent a kind of linearization beyond perturbation approach for nonlinear systems. For linear case with *g*_4_ = 0, the normal modes of Eq. (2) are recovered.

The existence of renormalized vibrations of Eq. (5) can be verified by numerical simulations. The recently developed resonance phonon approach method^53,54^, will be used to numerically calculate the dispersion relation of the renormalized vibration modes in DNLS models (See Methods for the brief introduction of the resonance phonon approach method). The equations of motions will be numerically integrated by symplectic algorithm to achieve high accuracy. Since the Hamiltonian of Eq. (3) cannot be separated into the form as *H*(*q*, *p*) = *T*(*p*) + *V*(*q*), the two-stage, fourth-order symplectic Gauss implicit Runge-Kutta method^55^ will be applied for numerical integrations.

**Figure 1:**
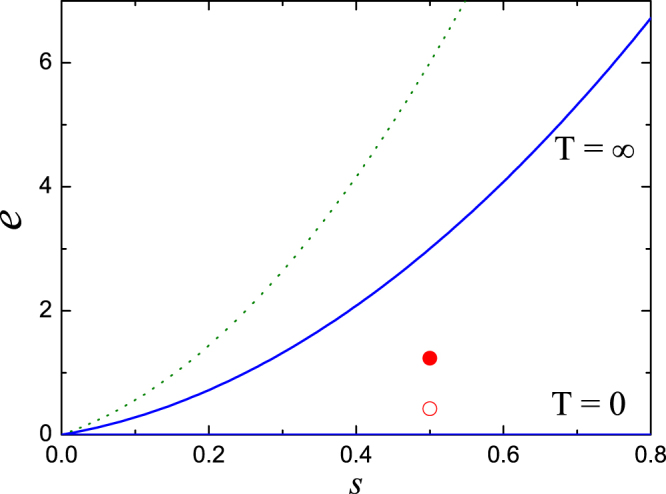
The parameter space (*e*, *s*) for FPU-*β*-like DNLS model with *g*_2_ = *g*_4_ = 1. The solid curves *e* = 0 and *e* = 2*g*_2_*s* + 8*g*_4_*s*^2^ correspond to the temperature *T* = 0 and *T* = ∞, respectively. The dotted curve *e* = 4*g*_2_*s* + 16*g*_4_*s*^2^ denotes the upper limit where numerical simulations can approach. The empty and filled circles are the two parameter setups used in Fig. 1 for FPU-*β*-like DNLS models.

In microcanonical numerical simulations, the input parameters are the energy density *e* = *H*/*N* and norm density *s* = *S*/*N*. There is a one-to-one correspondence between (*e*, *s*) and (*β*, *μ*) where *β* = (*k*_*B*_*T*)^−1^ is the inverse temperature and *μ* is the chemical potential, only if the grand-canonical partition function *Z* is meaningful^56^:

6 $$Z={\int }_{0}^{\infty }{\int }_{0}^{2\pi }\prod _{j}d{\varphi }_{j}d{s}_{j}{e}^{-\beta (H+\mu S)}$$

with the canonical transformation ${\psi }_{j}=\sqrt{{s}_{j}}{e}^{i{\varphi }_{j}}$ being applied. Following the ref.^56^, one can derive here that the zero and infinite temperature line *T* = 0 and *T* = ∞ will correspond to *e* = 0 and *e* = 2*g*_2_*s* + 8*g*_4_*s*^2^ in (*e*, *s*) parameter space (Fig. 1, *g*_2_ = *g*_4_ = 1), respectively. In our numerical simulations, the norm density is always chosen as *s* = 0.5 and the energy densities are chosen as some portions of the energy density limited by the infinite temperature. As discussed in ref.^56^, the region of the parameter space that is numerically accessible can be wider than these two limits. Actually in our numerical settings, the upper limit in parameter space is the line of *e* = 4*g*_2_*s* + 16*g*_4_*s*^2^ (the dotted line in Fig. 1 with *g*_2_ = *g*_4_ = 1).

**Figure 2:**
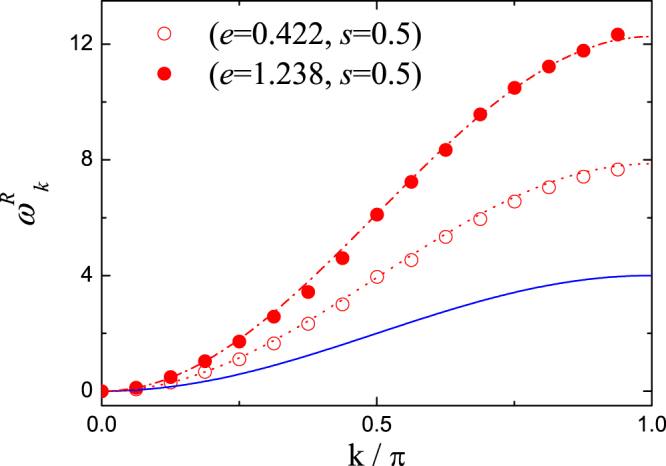
The renormalized vibrations in FPU-*β*-like DNLS model with size *N* = 32. The blue solid curve is the dispersion relation ${\omega }_{k}=4\,{\sin }^{2}(k\mathrm{/2})$ of Eq. (2) for linear system. The empty and filled circles are the numerical data of renormalized vibrations in (*e*, *s*) parameter spaces with (0.422, 0.5) and (1.238, 0.5). The dotted and dash-dotted curves are the renormalized dispersion relations ${\omega }_{k}^{R}=\alpha {\omega }_{k}$ from renormalization theory, where the renormalization coefficients *α*’s are calculated from Eq. (5) as *α* = 1.966 and *α* = 3.066, respectively. Perfect agreements between numerical simulations and renormalization theory are obtained.

In Fig. 2, the renormalized vibration frequencies directly calculated for a lattice size *N* = 32 with two parameter setups of (*e* = 0.422, *s* = 0.5) and (*e* = 1.238, *s* = 0.5) are plotted as empty and filled circles for the FPU-*β*-like DNLS model. From the renormalization theory, the renormalization coefficient *α* can be calculated via time averages of Eq. (5) as *α* = 1.966 and *α* = 3.066, respectively. The theoretically determined renormalized vibration frequencies ${\omega }_{k}^{R}=\alpha {\omega }_{k}$ are plotted as the dotted and dash-dotted lines in Fig. 2, and perfect agreements with direct numerical calculations are observed.

**Figure 3:**
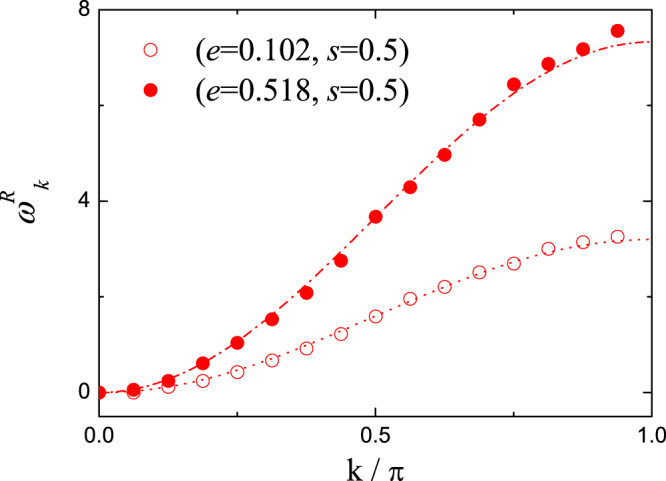
The renormalized vibrations in *H*_4_-like DNLS model with size *N* = 32. The empty and filled circles are the numerical data of renormalized vibrations in (*e*, *s*) parameter spaces with (0.102, 0.5) and (0.518, 0.5). These numerical symbols are compared with the renormalization theory results of ${\omega }_{k}^{R}=\alpha {\omega }_{k}$ with *α* = 0.780 (dotted) and *α* = 1.832 (dash-dotted), respectively. Here ${\omega }_{k}=4\,{\sin }^{2}(k/2)$ and perfect agreements with renormalization theory are obtained.

We then consider the pure nonlinear *H*_4_-like DNLS model with *g*_2_ = 0 and *g*_4_ = 1. The renormalized vibration frequencies are directly calculated for two parameter setups of (*e* = 0.102, *s* = 0.5) and (*e* = 0.518, *s* = 0.5) with size *N* = 32, as can be seen in Fig. 3 plotted as empty and filled circles. These numerical data are compared with the renormalization theory (dotted and dash-dotted lines in Fig. 3) of Eq. (5) with *α* being calculated as *α* = 0.780 and *α* = 1.832, respectively. It should be noticed that there is no linear term in the *H*_4_-like DNLS model. The perfect agreements between direct numerical calculations and renormalization theory are also obtained. These results demonstrate that the effective vibrational modes in nonlinear DNLS models can be well described by the generalized renormalization theory. The renormalized vibration effect due to nonlinearity should be a more generalized phenomenon in non-integrable systems.

As we have mentioned in the introduction part, all the 1d nonlinear momentum-conserving atomic lattices including FPU-*β* and *H*_4_ lattices have anomalous energy transport, with the coupled rotator lattice as an exception. The energy transport property can be investigated by the energy diffusion processes via the spreading of the spatio-temporal correlation function of the local excess energy defined as^57,58^:

7 $${\rho }_{E}(j,t)=\frac{\langle {\rm{\Delta }}{H}_{j}(t){\rm{\Delta }}{H}_{0}(0)\rangle }{\langle {\rm{\Delta }}{H}_{0}(0){\rm{\Delta }}{H}_{0}(0)\rangle }+\frac{1}{N-1}$$

where Δ*H*_*j*_(*t*) = *H*_*j*_(*t*) − 〈*H*_*j*_〉 and *H*_*j*_ = *g*_2_|*ψ*_*j*+1_ − *ψ*_*j*_|^2^+*g*_4_|*ψ*_*j*+1_ − *ψ*_*j*_|^4^ is the local energy. The small constant value 1/(*N* − 1) is introduced to ensure the normalization condition ∑_*j*_*ρ*_*E*_(*j*, *t*) = 1 in microcanonical simulations^58^. In thermodynamical limit *N* → ∞, this term will vanish and microcanonical and canonical systems are equivalent as expected. For simplicity, the periodic boundary conditions *ψ*_*j*_ = *ψ*_*j*+*N*_ are used. The index *j* is assigned as −*N*/2 + 1, ..., −1, 0, 1, ..., *N*/2, where the lattice size *N* is set as an even number for convenience.

**Figure 4:**
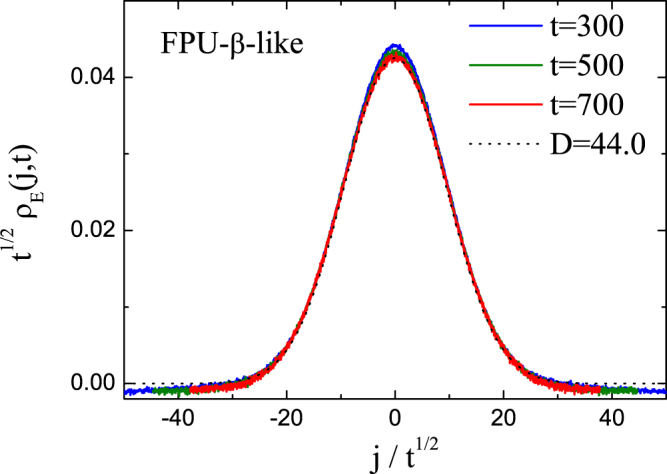
The rescaled local excess energy distribution function *ρ*_*E*_(*j*, *t*) for FPU-*β*-like DNLS model with *N* = 2000. The dotted curve is the normalized Gaussian distribution function with diffusion constant *D* = 44.0.

The energy correlation function *ρ*_*E*_(*j*, *t*) asymptotically approaches to a Levy walk distribution for anomalous transport and Gaussian distribution for normal transport^57–60^. The Gaussian distribution function is given by ${\rho }_{E}^{G}(j,t)=\frac{1}{\sqrt{4\pi Dt}}{e}^{-\frac{{j}^{2}}{4Dt}}$, where *D* is the diffusion constant. In Fig. 4, we plot the rescaled local excess energy distribution functions *ρ*_*E*_(*j*, *t*) for the FPU-*β*-like DNLS model with parameter setup of (*e* = 1.238, *s* = 0.5) for a size *N* = 2000. All the rescaled curves of *ρ*_*E*_(*j*, *t*) at three different correlation times *t* = 300, 500, 700 collapse into the same curve of the normalized Gaussian distribution with diffusion constant *D* = 44.0. This is a clear signature of normal energy transport. As we know the Mean Square Displacement (MSD) 〈Δ*x*^2^(*t*)〉_*E*_ measuring energy diffusion is defined as 〈Δ*x*^2^(*t*)〉_*E*_ ≡ ∑_*j*_ *j*^2^*ρ*_*E*_(*j*, *t*)^57–60^. The Gaussian disbrituion of *ρ*_*E*_(*j*, *t*) will give rise to the normal diffusion behavior as ${\langle {\rm{\Delta }}{x}^{2}(t)\rangle }_{E}={\sum }_{j}\,{j}^{2}{\rho }_{E}^{G}(j,t)\propto t$. This is different from the anomalous energy transport of 1d FPU-*β* and *H*_4_ lattices which exhibits superdiffusion behavior for energy diffusion as 〈Δ*x*^2^(*t*)〉_*E*_ ∝ *t*^*β*^ with 1 < *β* < 2^57–60^.

**Figure 5:**
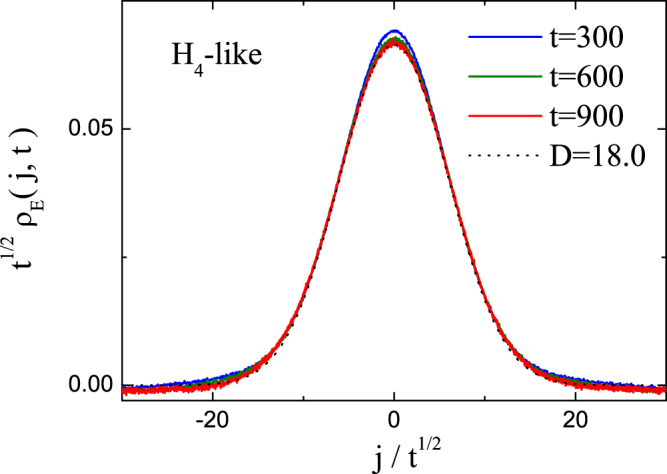
The rescaled local excess energy distribution function *ρ*_*E*_(*j*, *t*) for *H*_4_-like DNLS model with *N* = 2000. The dotted curve is the normalized Gaussian distribution function with diffusion constant *D* = 18.0.

In Fig. 5, the rescaled distribution functions *ρ*_*E*_(*j*, *t*) for the *H*_4_-like DNLS model are plotted with the parameter setup of (*e* = 0.518, *s* = 0.5). The dashed curve is the normalized Gaussian distribution function with diffusion constant *D* = 18.0. It can be seen that all the three rescaled *ρ*_*E*_(*j*, *t*) at time *t* = 300, 600, 900 collapse into the same curve described by the Gaussian normal distribution. Normal energy transport is always found for the 1d FPU-like DNLS models with inter-site nonlinearities, strikingly different from their atomic lattice counterparts such as the 1d FPU-*β* and *H*_4_ lattices.

## Discussion

For 1d nonlinear atomic lattices such as the FPU-*β* lattice with only inter-site nonlinearity, they have three conserved quantities: the total energy, total momentum and length (stretch)^40^. While for 1d nonlinear atomic lattices such as the FK and *ϕ*^4^ lattices with only on-site nonlinearity, only two of them are conserved due to the breaking of total momentum. Although the FPU-like DNLS models with inter-site nonlinearities are quite different with the DNLS models with on-site nonlinearities, they do have the same number of conserved quantities: the total energy and the total norm^35,36^. This is because that the norm conservation is due to the gauge invariance and related to the overall phase rotations^61^. It has nothing to do with the actual types of nonlinearities. As a result, both DNLS models with inter-site or on-site nonlinearities share the same conserved quantities. This might be the reason that both DNLS models exhibit normal energy transport behaviors.

For nonlinear systems with normal energy transport, the Hamiltonian of FPU-like DNLS models with two conserved quantities shares no similarity with that of atomic lattices such as FK and *ϕ*^4^ lattice with the same number of conserved quantities. However, the Hamiltonian of DNLS models do resemble that of the 1d coupled rotator lattice with three conserved quantities^6,7^:

8 $$H({\varphi }_{j},{p}_{j})=\sum _{j}[\frac{{p}_{j}^{2}}{2}+(1-\,\cos \,({\varphi }_{j+1}-{\varphi }_{j}))]$$

where *ϕ*_*j*_ and *p*_*j*_ are the generalized canonical coordinates and momenta, respectively. The coupled rotator lattice conserves the total energy, total momentum and stretch as that of FPU-*β* lattice.

If we notice that under the canonical transformation of ${\psi }_{j}=\sqrt{{s}_{j}}{e}^{i{\varphi }_{j}}$, the DNLS model of Eq. (1) can be transformed into the following expression:

9 $$\begin{array}{rcl}H({\varphi }_{j},{s}_{j}) & = & \sum _{j}[({g}_{2}+{g}_{4}({s}_{j}+{s}_{j+1}))({s}_{j}+{s}_{j+1})\\  &  & -\,\mathrm{(2}{g}_{2}+4{g}_{4}({s}_{j}+{s}_{j+1}))\sqrt{{s}_{j}{s}_{j+1}}\,\cos ({\varphi }_{j+1}-{\varphi }_{j})\\  &  & +\,4{g}_{4}{s}_{j}{s}_{j+1}{\cos }^{2}({\varphi }_{j+1}-{\varphi }_{j})]\end{array}$$

where *s*_*j*_ = |*ψ*_*j*_|^2^. It can be seen that both the coupled rotator lattice of Eq. (8) and the DNLS model of Eq. (9) share the same invariant property under the transformation of *ϕ*_*j*_ → *ϕ*_*j*_ + 2*π*. Although they have different number of conserved quantities, they might have the same mechanism for the normal energy transport.

In conclusion, we have investigated the dynamics in 1d FPU-like DNLS models with inter-site nonlinearities. The renormalization theory has been developed for the DNLS models which is verified by the numerical simulations. It has been found that the 1d FPU-like DNLS models exhibit normal energy transport strikingly different from their atomic lattice counterparts such as the 1d FPU-*β* lattice. The FPU-like DNLS models with inter-site nonlinearities have the same normal energy transport as the DNLS models with on-site nonlinearities since they have the same two conserved quantities of total energy and total norm, where the latter is due to the gauge transformation. However, this normal energy transport in DNLS models resembles that of 1d coupled rotator lattice where both models share a same invariant property. The two typical nonlinear systems studied in different physical areas might have the same physical mechanism for the normal energy transport. It has been proved that the DNLS models with the form of Eq. (1) cannot preserve the total momentum defined as $M\equiv i\sum ({\psi }_{j+1}^{\ast }{\psi }_{j}-{\psi }_{j+1}{\psi }_{j}^{\ast })$^62^. It will also be interesting to study the transport property of a new DNLS model with a third conserved quantity.

## Methods

The resonance phonon approach method is intended to numerically calculate the renormalized phonons ${\omega }_{k}^{R}$ in 1d nonlinear atomic lattices^53,54^. But it turns out that this novel method is able to calculate the renormalized vibrations in general 1d nonlinear lattices including the DNLS models. Considering the DNLS Hamiltonian *H*(*q*_*j*_, *p*_*j*_) of Eq. (3), we need first calculate the equilibrium-state correlation function between the *m*-th and *n*-th sites:

10 $${{\rm{\Phi }}}_{mn}(t)=\langle {q}_{n}(0){q}_{m}(t)\rangle $$

here 〈⋅〉 denotes the ensemble average which is equivalent to time average if the system is chaotic. Since the dynamical variables *q*_*m*_ and *p*_*m*_ are symmetrical here, one can also calculate the correlation function for *p*_*m*_. As the system is translationally invariant, the correlation function Φ_*mn*_(*t*) depends on their relative distance *j* = *m* − *n* only.

The spatial Fourier transform of the correlation function is then performed as

11 $$\varepsilon (k,t)=\sum _{j}{{\rm{\Phi }}}_{j}(t){e}^{-ikj}$$

the space mode *ε*(*k*, *t*) contains only real part since Φ_−*j*_(*t*) = Φ_*j*_(*t*). The real Φ_*j*_(*t*) also ensures the mirror symmetry for *ε*(*k*, *t*) as *ε*(−*k*, *t*) = *ε*(*k*, *t*). One can simply consider the positive mode *k*. The space mode *ε*(*k*, *t*) oscillates with time and we can do another temporal Fourier transform as:

12 $$\chi (k,\omega )={\int }_{0}^{\infty }\varepsilon (k,t){e}^{i\omega t}dt$$

this so-obtained *χ*(*k*, *ω*) is called wave-number-dependent complex response function. It contains a real and an imaginary part as

13 $$\chi (k,\omega )=\chi ^{\prime} (k,\omega )+i\chi ^{\prime\prime} (k,\omega )$$

Both *χ*′(*k*,*ω*) and *χ*′′(*k*, *ω*) can give the information for the renormalized vibrations. For each mode *k*, the *χ*′(*k*, *ω*) achieves its maximum value at some frequency *ω*_*max*_, which is identified as the renormalized vibration frequency ${\omega }_{k}^{R}$ for the mode *k* as can be seen from Fig. 2(a) in ref.^54^. The dispersion relation of renormalized vibrations ${\omega }_{k}^{R}$ can thus be numerically obtained via this novel resonance phonon approach method^53,54^.
